# The Effect of Various Media and Hormones via Suspension Culture on Secondary Metabolic Activities of (Cape Jasmine) *Gardenia jasminoides* Ellis

**DOI:** 10.1155/2014/407284

**Published:** 2014-05-21

**Authors:** Reza Farzinebrahimi, Rosna Mat Taha, Kamaludin Rashid, Jamilah Syafawati Yaacob

**Affiliations:** ^1^Institute of Biological Sciences, Faculty of Science, University of Malaya, 50603 Kuala Lumpur, Malaysia; ^2^Biology Division, Centre for Foundation Studies in Science Building, University of Malaya, 50603 Kuala Lumpur, Malaysia

## Abstract

The leaf of *Gardenia jasminoides* Ellis was used as explants and was cultured on MS and WPM media supplemented with various concentrations of NAA, IAA, 2,4-D, IBA, TDZ, and Kn (0 to 5 mg L^−1^ with 0.5 increment). After six months, the higher percentage of callus (100%) and the best dry and fresh weight of callus were formed on WPM medium supplemented with 2,4-D and NAA (2.0-3.0 mg L^−1^) and this amount was decreased from (84%) to (69%) when this media supplemented with Kinetin and TDZ (1 mg L^−1^) respectively were used. Leaf segments cultured on WPM media added with Kn (1 mg L^−1^) and TDZ (2 mg L^−1^) yielded the least amount of callus. It was found that WPM media added with IAA (4.5–5.0 mg L^−1^) were optimum for root induction from *G*. *jasminoides* plantlets. Antibacterial screening of leaf extracts (*in vivo*) showed no inhibitory effect against *E. coli*, *P. aeruginosa*, *S. aureus,* and *B. cereus*, in contrast to callus extracts from leaf cultures supplemented with NAA, which showed inhibition activity against *E. coli* and *B. cereus*. The callus extracts from leaf cultures grown on both MS and WPM media showed higher antioxidant and superoxide dismutase activities than leaf extracts.

## 1. Introduction

*Gardenia jasminoides* Ellis (Cape jasmine) belongs to Rubiaceae family and is native to the tropical and subtropical parts of Africa, Southern Asia, Australia, and Oceania. Gardenias are widely practiced as an ornamental flower in bouquets, as houseplants, and as outside plants. This plant is known as one of the most valuable plant species in traditional Chinese medicine and is considered highly effective as a haemostatic agent, drain fire, and is effective in treating injuries to the muscles, joints, and tendons [1]. The major components of Gardenia's fruits are iridoid glycosides, which can be converted into blue and red pigments. These crocetin derivatives are known for their colouring properties and their particular water-soluble behavior, in contrast to most plant families of carotenoids [2]. Carotenoids are thought equally as the main contributors to the antioxidant content of a particular plant and have been proven to yield beneficial pharmacological effects, such as preventing cardiovascular diseases [3].

Benzylaminopurine (BAP) gave high proliferation of the* G. jasminoides* compared to 6-*γ*-*γ*-[dimethylallylamino]-purine (2iP) and kinetin [4], while using indole-3-butyric acid (IBA) in micro cuttings of* G. jasminoides* showed high percentages of root* in vitro* and* ex vitro* [5, 6]. A combination of (TDZ) thidiazuron (1.7 mg L^−1^) with (IAA) indole-3-acetic acid (1 mg L^−1^) produced adventitious shoots on* Gardenia* explants [7]. Free radical scavenging activity has been reported from* Gardenia jasminoides* fruit extracts [8], as well as leaf extracts when grown* in vivo* and* in vitro* [9].* G. jasminoides* also showed superoxide dismutase-like (SOD-like) activity [10]. The use of plant extracts and phytochemicals with antimicrobial contents can be of great significance and, recently, a number of studies have proven such efficiency. Izzo et al. [11] revealed the effect of phytochemical and the antimicrobial activity of anacardic acid which had an inhibitory effect on* Staphylococcus aureus*,* Brevibacterium ammoniagenes*,* Streptococcus mutans,* and* Propionibacterium acnes*. The present investigation aimed to study the antibacterial and antioxidant properties of* G. jasminoides* extracts obtained from leaves (*in vivo*) and callus (*in vitro*). For callus induction, leaf explants were cultured on MS and WPM media supplemented with various hormones at different concentrations. This study differs from previous work in the sense that callus or undifferentiated cells from* in vitro* or tissue culture system were used for comparison with intact tissue (*in vivo*).

## 2. Materials and Methods

### 2.1. Plant Materials

Six-month-old, young, and fresh leaves of* G. jasminoides* Ellis obtained from Serdang nursery (Malaysia) were surface sterilized by using mercuric chloride, Clorox, and ethanol [7, 9]. The sterilized explants were cut into 50 × 50 mm segments and cultured onto 60 mL Murashige and Skoog (MS) medium [12] and WPM [13] in a sterile container, supplemented with various concentrations of auxins (NAA, IAA, IBA, and 2,4-D or 2,4-dichlorophenoxyacetic acid), cytokinins (TDZ, Kn) (0.5–5.0 mg L^−1^), sucrose (30 g), and Gelrite (5 g). The pH was maintained on 5.7 ± 2 using HCL and NaOH (1 N). The 3-month callus formed* in vitro* was tested by double staining method [14] to obtain embryogenic cells. The embryogenic callus (50 g) was weighed out in laminar flow cabinet and was inoculated on the same hormone concentrations and media without agar and maintained on a horizontal shaker (120 rpm) in a growth chamber at 24°C, with 16 hours light and 8 hours dark conditions.

The callus aggregate was used as samples for subsequent analysis after 6 months of culture. Young leaves of intact* G. jasminoides* (60 g) were also used as a comparison (*in vivo*).

### 2.2. Extract Preparation

*In vitro* samples (6-month-old callus) and* in vivo* samples (leaves of intact* G. jasminoides*) were dried in an incubator at 40°C and ground to produce fine homogenous powder by using an electric blender. The ground powders (3 g) of both samples were soaked in 5% Tween 20 (40 mL) at room temperature and were filtered through Whatman No. 1 filter paper (Whatman International, England).

### 2.3. Antioxidant Activity Assay

Superoxide dismutase (SOD) determination kit (19160), ascorbic acid (A4544) from Sigma-Aldrich (St. Louis, Mo), and tert-butylated hydroxytoluene (34750) from Fluka (Spain) were purchased and used for this part of the study. Plant extracts (20 *μ*L) with a concentration of 10 g L^−1^ were added to 200 *μ*L of the kit working solution. The mixture, after a gentle shaking, was incubated at 37°C for 20 min after adding 20 *μ*L of the kit enzyme working solution. The absorbance of the mixtures was measured at 450 nm using a microplate reader (BIO-RAD Model 550, USA) and the SOD activity was calculated based on the following equation [15]. Ascorbic acid (1 g L^−1^) and BHT or tert-butylated hydroxytoluene (1 g L^−1^) were employed as the positive controls in this study:

(1) Inhibition%(SOD  activity) =(blank1−blank2)−(sampleA−blanksampleA)(blank1−blank2)×100,

where blank_1_ = blank of mixture working solution + enzyme working solution + double distilled water, blank_2_ = blank of mixture plant extract + working solution + dilution buffer + double distilled water, and blank_sample_A__  = blank of mixture plant extract + working solution + dilution buffer.

### 2.4. Antibacterial Activity Assay

The antibacterial potential of* G. jasminoides* was studied based on the paper disc diffusion method [15, 16]. Two gram-negative pathogenic bacteria (*Escherichia coli* and* Pseudomonas aeruginosa*) and two gram-positive bacteria (*Bacillus cereus* and* Staphylococcus aureus*) were obtained from Microbiology Division of Institute of Biological Sciences and grown in a nutrient broth medium to yield a final concentration of 107 colony forming unit (CFU) per mL^−1^. The test bacteria (0.1 mL) were streaked on Mueller Hinton medium (MH) plates using sterile cotton swab. Sterilized filter paper discs were soaked in extracts (100 g L^−1^) and then placed at the center of test bacteria plates. The plates were incubated for 24 h and the diameters of the inhibition zones were measured. Tetracycline disc (30 *μ*g) and PBS were used as the positive and negative controls, respectively.

### 2.5. Statistical Analysis

Experiments were designed based on complete randomized design (CRD) with six replicates. Data analysis was carried out using SPSS version 21 and analysis of variance (ANOVA) was carried out. The means were separated via Duncan's multiple comparison test (DMCT) and *P* < 0.05 was considered to indicate statistical significance.

## 3. Results

### 3.1. Induction of Callogenesis from Leaf Explants of* Gardenia jasminoides*

Initiation of callus was observed from the young leaf explants after three weeks of culture on the MS and WPM medium supplemented with different plant growth regulators such as NAA and IAA. The friable, greenish, and yellowish callus were formed on the media supplemented with TDZ (1 and 1.5 mg L^−1^) and IBA (1 mg L^−1^). The results of double staining test showed that embryogenic cells were formed on callus grown with various auxin and nonembryogenic callus formed on MS supplemented with TDZ and Kn.

**Figure 1:**
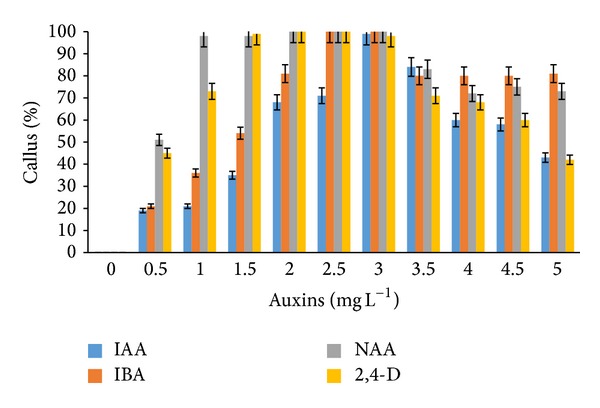
Percentage of* callus formation* from* leaf explants* of* G. jasminoides* cultured on* MS media* supplemented with different types of* auxin* at various concentrations after 6 months of culture (*P* < 0.05, *n* = 6).

**Figure 2:**
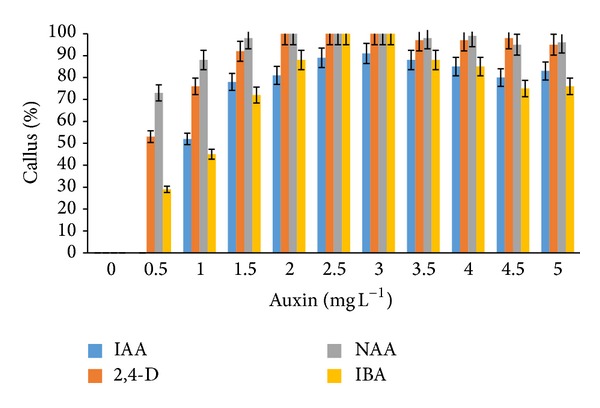
Percentage of* callus formation from leaf explants* of* G. jasminoides* cultured in* WPM medium* supplemented with different types of* auxin* at various concentrations after 6 months of culture (*P* < 0.05, *n* = 6).

**Figure 3:**
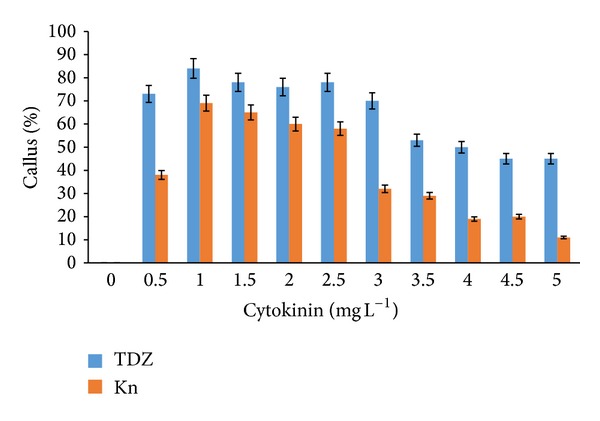
Percentage of* callus formation from leaf explants* of* G. jasminoides* cultured on* MS media* supplemented with different types of* cytokinin* at various concentrations after 6 months of culture (*P* < 0.05, *n* = 6).

**Figure 4:**
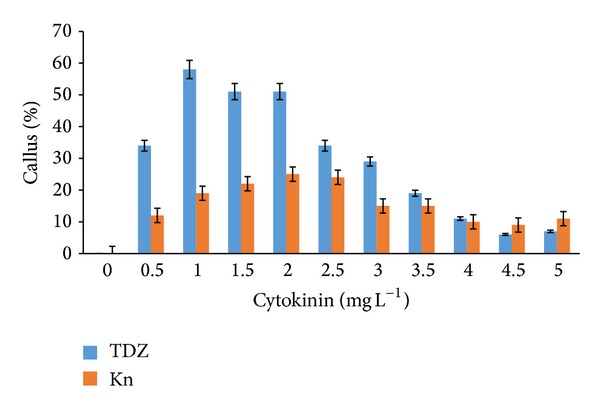
Percentage of* callus formation from leaf explants* of* G. jasminoides* cultured in* WPM medium* supplemented with different types of* cytokinin* at various concentrations after 6 months of culture (*P* < 0.05, *n* = 6).

**Figure 5:**
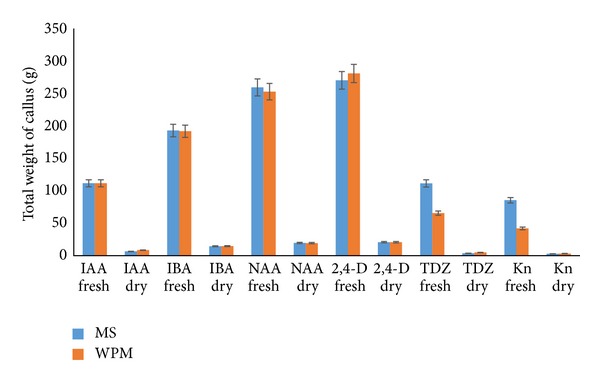
*Mean comparison between dry and fresh weight of callus* obtained from leaf explants of* G. jasminoides* cultured on WPM and MS media supplemented with various hormones (*P* < 0.05, *n* = 6).

Callus formation was observed after as early as three to five weeks of induction on both MS and WPM media supplemented with auxins and cytokinins. MS media supplemented with IBA and NAA (2.0–3.0 mg L^−1^) as well as IAA (2.5–3.0 mg L^−1^) also showed 100% callus formation. However, the addition of 2,4-D (2.5–3.0 mg L^−1^) to the MS media only produced 90% callus formation (Figure 1). Extremely high callus percentage (100%) was observed from WPM media supplemented with NAA and 2,4-D (2.0–3.0 mg L^−1^) alone and IBA (2.5–3.0 mg L^−1^) (Figure 2). Lower portions of callus growth (84% and 69%) were observed when WPM media were supplemented with kinetin and TDZ (1.0 mg L^−1^), respectively (Figure 3). However, the addition of kinetin and TDZ to WPM was found to be ineffective as shown by the striking decrease in callus formation to 58% and 25% when kinetin (1.0 mg L^−1^) and TDZ (2.0 mg L^−1^) were added alone, respectively (Figure 4). In contrast, adding kinetin and TDZ to MS media was found to be beneficial and helped boost callogenesis in* G. jasminoides*. It was found that both media supplemented with 2,4-D and kinetin affected the callus weight. The type of media also influenced the yield of callus when supplemented with similar plant hormones. It was perceived that the addition of 2,4-D (3.0 mg L^−1^) and kinetin (5.0 mg L^−1^) to the WPM media yielded 34.23 g and 3.39 g of fresh and dry weight of callus, respectively. In contrast, MS media supplemented with 2,4-D (2.5 mg L^−1^) and kinetin (4.0 mg L^−1^) only generated 30.04 g and 3.78 g fresh and dry weight of callus, respectively (Figure 5).

**Figure 6:**
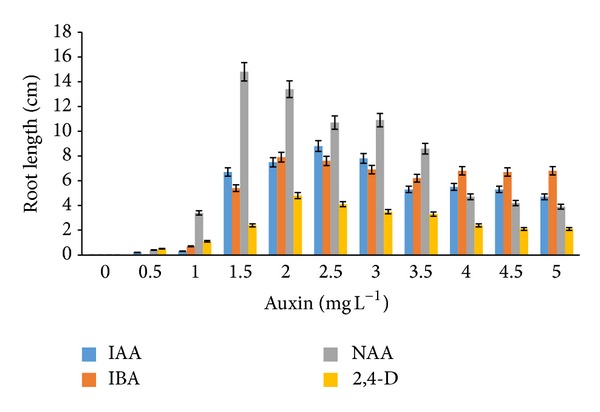
*Root length* of* G. jasminoides* when cultured on* MS medium* supplemented with various* auxins* after 6 months of culture (*P* < 0.05, *n* = 6).

**Figure 7:**
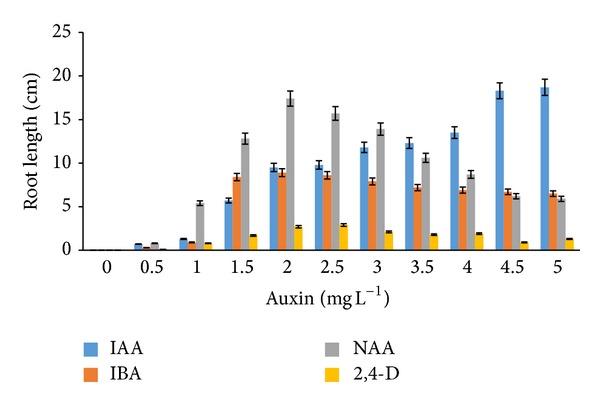
*Root length* of* G. jasminoides* when cultured in* WPM medium* supplemented with various* auxins* after 6 months of culture (*P* < 0.05, *n* = 6).

Rooting induction and root elongation from* in vitro* regenerates of* G. jasminoides* were also examined in the present investigation. The addition of auxins to both MS and WPM media demonstrated different results. Rooting was observed from leaf explants cultured on both MS and WPM media after the fifth week. In WPM medium, the use of NAA (1.5–2.0 mg L^−1^) exhibited excellent root growth of 14.8 and 13.4 cm, respectively (Figure 6). Addition of 2,4-D (4.5–5.0 mg L^−1^) and NAA (2.0–3.0 mg L^−1^) to WPM media also promoted root growth (Figure 7).

### 3.2. Screening Antibacterial Activity of the Leaf and Callus Extracts in* Gardenia jasminoides*

Antibacterial activity was determined by measuring the zone of inhibition depicted by leaf and callus extracts of* G. jasminoides* extracts on* Escherichia coli*,* Staphylococcus aureus, Pseudomonas aeruginosa,* and* Bacillus cereus*. It was found that leaf extracts of* G. jasminoides* grown* in vivo* showed no inhibitory activity against all four types of bacteria (Table 1). In contrast, callus extracts grown on both MS and WPM media supplemented with NAA showed inhibitory effects on* Escherichia coli* and* Bacillus cereus* (Table 1). However, no inhibitory influence was observed by* G. jasminoides* callus extracts against* Staphylococcus aureus* and* Pseudomonas aeruginosa* (Table 1).

### 3.3. Screening for Antioxidant and Superoxide Dismutase Activities

**Figure 8:**
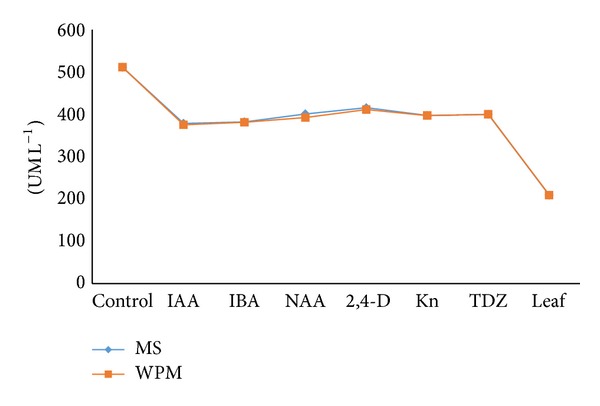
A comparison of superoxide dismutase (SOD) activity between leaf extracts of* in vivo* grown plant and callus extracts from leaf cultures of* G. jasminoides* grown on different media (MS and WPM) supplemented with various plant hormones (*P* < 0.05, *n* = 5).

Antioxidant including superoxide dismutase activities was evaluated via superoxide dismutase (SOD) kit. Analysis of results showed that callus extracts of* G. jasminoides* (~30 U mL^−1^) had significantly higher superoxide dismutase activities compared to leaf extracts of* in vivo* (10 U mL^−1^) grown* G. jasminoides* (Figure 8). The addition of various plant hormones to the callus regeneration media revealed no significant effect in influencing the antioxidant and superoxide dismutase activities expressed by specific callus extracts (Figure 8). These data recommend that plant hormones performed a role in modifying antioxidant and superoxide dismutase properties of a plant, although not significant in* G. jasminoides*. The present study also proves that antioxidant and superoxide dismutase potential of a plant (such as* Gardenia jasminoides* Ellis) can be improved by means of tissue culture, particularly via callogenesis.

## 4. Discussion

In the current work, callus was obtained from MS and WPM media supplemented with all different kinds of hormones. However, embryonic callus was only formed on the media that were supplemented with various types of auxin. The greenish and yellowish callus observed on both media supplemented with TDZ and IBA were similar to those reported by Eeckhaut et al. [17] on this family.

Using leaf segments from* G. jasminoides* plants germinated* in vitro*, friable calli were induced by the presence of cytokinins. This coincides with results obtained by Al-Juboory et al. [7] who reported shoot induction from leaf explants in MS media supplemented with TDZ and Kn (0.5 mg L^−1^). In this investigation, no shoot formation was observed from leaf cultures of* G. jasminoides* when TDZ and kinetin were added to the growth media, contrary to the findings by Duhoky and Rasheed [18] and Sayd et al. [9] who reported the formation of shoots from leaf cultures of this species.

The present study was conducted to mass propagate* G. jasminoides* Ellis via* in vitro* techniques by utilizing different growth media and various plant hormones and it also aimed to manipulate and evaluate antibacterial, antioxidant, and superoxide dismutase properties of* G. jasminoides* via callogenesis. Based on the results obtained from this investigation, it was observed that callus can be readily induced from both types of media (MS and WPM), simply by increasing the plant hormones which may exhibit different effects when supplemented to different growth media. Concentration of plant hormones was similarly used to affect the percentage of callus formation.

2,4-D has been the best option of auxin and has been widely utilized to promote callus induction in many plant species. However, the present study revealed that WPM media supplemented with this hormone produced an inadequate performance in callogenesis of* G. jasminoides* plants as shown by the low values of both fresh and dry weights of callus obtained. Abdel-Rahim et al. [19] and Sayd et al. [9] reported the effect of 2,4-D when added to MS media.

The data analyzed showed no significant differences in callus formation between various auxins on WPM media; in contrast, MS media showed significant differences between IAA to other auxins. In addition, kinetin and TDZ supplemented to MS media showed statistically significant differences compared to WPM media. There was little difference between these two hormones on WPM media. More variations of callus formation calculated on WPM supplemented with TDZ compare to Kn.

MS media have been widely utilized in tissue culture of Cape jasmine [7, 9, 18]; WPM media were found to be a preferable choice, especially for callogenesis, due to its different nutrient, calcium, and phosphate content [20].

This study also investigates the antibacterial properties of* G. jasminoides* plant extracts, especially from leaf extracts of* in vivo* grown plants as well as callus extracts of this species. In general, plant extracts are usually more active and exhibited higher inhibitory influence against gram-positive (*S. aureus*) bacteria compared to gram-negative bacteria (*S. typhimurium*) [21]. Abu-Shanab et al. [22] reported higher resistance of gram-negative bacteria to plant extracts compared to gram-positive bacteria. This is probably due to the different permeability of the bacterial cell wall or membrane accumulation process [23]. In the present study, experimental data demonstrated that leaf extracts of* in vivo* grown* G. jasminoides* showed no inhibition against four tested bacteria. However, callus extracts obtained from leaf cultures grown on both MS and WPM media supplemented with NAA revealed inhibitory impression against one gram-negative and one gram-positive bacteria, specifically* Escherichia coli* and* Bacillus cereus*. These results are in agreement with the previous findings by Wei et al. [23], Abdul Manaf [24], Møretrø et al. [25], Ragasa et al. [26], and Wei et al. [27]. Data analysis also recommended that antibacterial properties of a plant (such as* Gardenia jasminoides*) could be manipulated to be improved through tissue culture procedures, especially via callogenesis. This might be due to the use of plant hormones that may modify or have an effect on the synthesis of bioactive compounds of* G. jasminoides*.

Moreover, antioxidant and superoxide dismutase activities were also studied in the present investigation. Superoxide dismutase (SOD) is an enzyme that catalyzes the dismutation of superoxide into oxygen and hydrogen peroxide. As such, SOD performed an essential role in the cell's antioxidant defense against oxygen. SOD is also a portion of the defense system in aerobic organisms against oxidative stress, especially in catalysis of superoxide anion (O_2_) and hydrogen peroxide, which are reduced to water via hydrogen peroxide scavenging enzyme (catalase). Hence, catalase and SOD are considered to limit the accumulation of reactive oxygen. SOD kit-like enzymes have been used by Miszalski et al. [28], Dos Santos et al. [29], and Shilpashree and Kumar [30] to investigate antioxidant activities exhibited by different species. Antioxidant properties had also been reported from* G. jasminoides* fruit extracts [27, 31–33]. Up to our knowledge, the present investigation reports for the first time the manipulation and enhancement of antioxidant properties of* G. jasminoides* through tissue culture (callogenesis) by using plant hormones such as IBA, TDZ, and kinetin to enhance growth and promote the synthesis of bioactive compounds of* G. jasminoides*. The results obtained in the present investigation proved that* Gardenia jasminoides* is a rich source of antioxidants, and the use of plant hormones can improve superoxide dismutase potential and increase antibacterial properties of this plant against* Escherichia coli* and* Bacillus cereus*.

WPM medium was observed to be a better medium for callus formation from* G. jasminoides* leaf explants compared to MS medium, possibly due to its different nutrient (calcium or phosphate) content.* G. jasminoides* was discovered to be rich in antioxidants and its antioxidant properties are improved* in vitro* via the utilization of plant hormones. Antioxidant properties exhibited by callus extracts of these species showed statistical difference compared to leaf extracts from* in vivo* grown plant. The callus extracts obtained from growth media supplemented with NAA showed antibacterial activity against* Escherichia coli* and* Bacillus cereus*, while leaf extracts from* in vivo* grown* G. jasminoides* showed no inhibitory effect against all tested bacteria. Alternative growth media can also be utilized for micropropagation of* G. jasminoides* such as SH, B5, and LS media. Antitumor and other biological activity tests of* G. jasminoides* extracts can also be investigated in the near future.

## Supplementary Material

Callus was formed in different shapes and colours when leaf explant of G.jasminoides cultured on various media and hormones. The root elongation was varied on media and hormones. However the result double staining test shows as embryonic heads stained red (acetocarmine) and *‎* suspensors stained blue (Evan's blue).

## Figures and Tables

**Table 1:** Inhibition zone exhibited by leaf extracts (100 g L^−1^) *in vivo* grown and callus extracts (*in vitro*) from leaf cultures of *Gardenia jasminoides* against four pathogenic bacteria (*n* = 5).

Bacteria	Inhibition zone (mm)		
	*In vivo* (leaf extracts)	*In vitro* (callus extracts from MS and WPM media supplemented with NAA)	Tetracycline (30 *μ*g)
*Escherichia coli *	−	+	39 ± 3.04
*Staphylococcus aureus *	−	−	21 ± 1.98
*Pseudomonas aeruginosa *	−	−	11 ± 1.99
*Bacillus cereus *	−	+	41 ± 3

−: no activity.

+: presence of activity.
