# Supplementary material for: The Effect of Various Media and Hormones via Suspension Culture on Secondary Metabolic Activities of (Cape Jasmine) Gardenia jasminoides Ellis
Source: ScientificWorldJournal. 2014 May 21;2014:407284. doi: 10.1155/2014/407284 (PMC4055353; doi:10.1155/2014/407284)
Supplement: Supplementary file 1 — Callus was formed in different shapes and colours when leaf explant of G.jasminoides cultured on various media and hormones. The root elongation was varied on media and hormones. However the result double staining test shows as embryonic heads stained red (acetocarmine) and ‎ suspensors stained blue (Evan's blue). [file 407284.f1.docx]

**
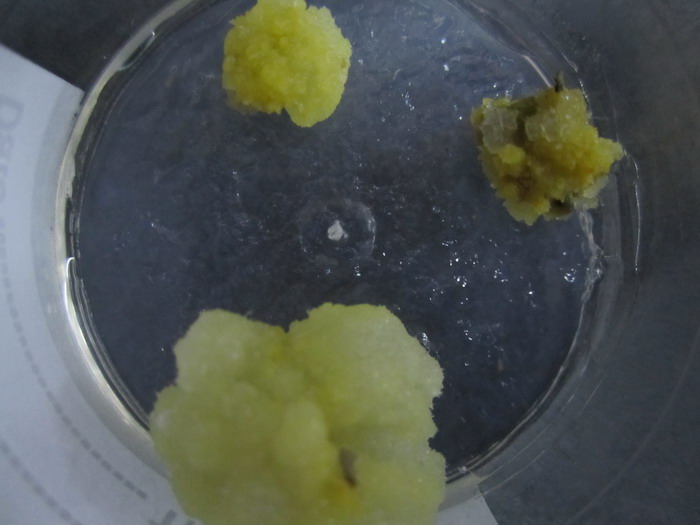

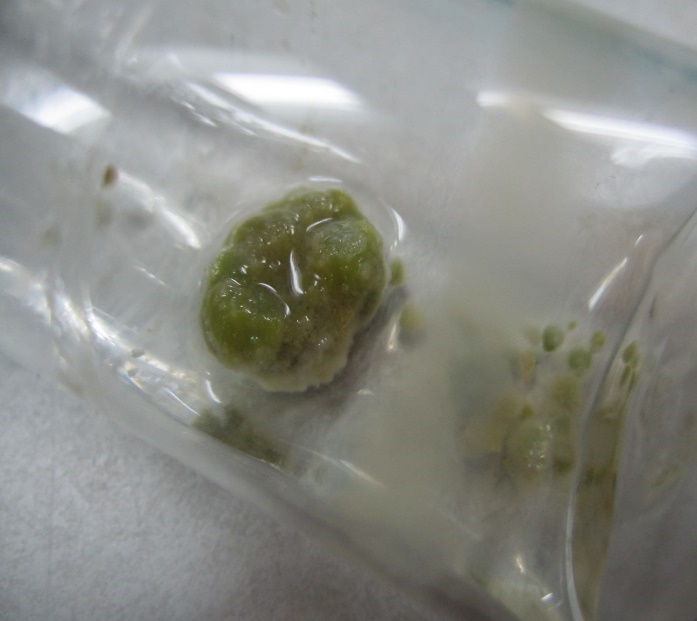
**

Greenish (right) and yellowish (left) callus from lead explant of *G.jasminoides* formed on WPM media supplemented with (1 mg L^-1^) TDZ and (1.5 mg L^-1^) IBA after 8 weeks


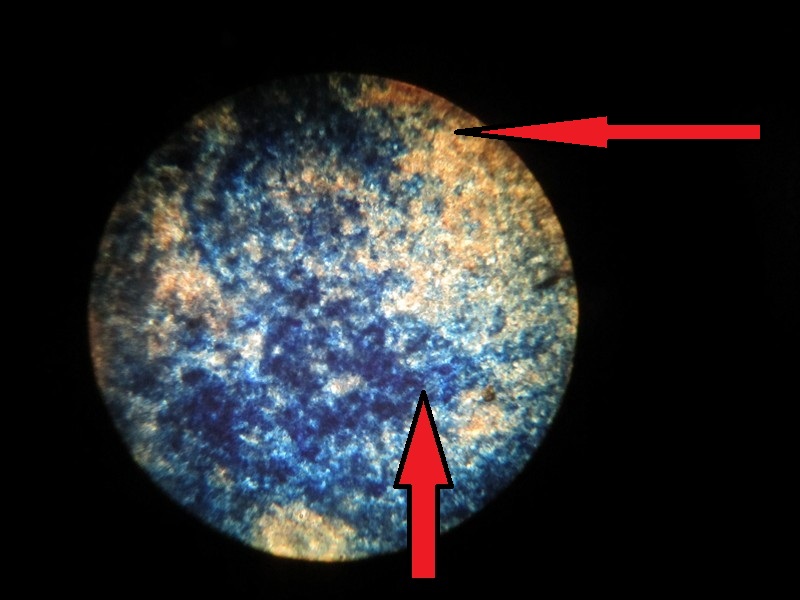
**
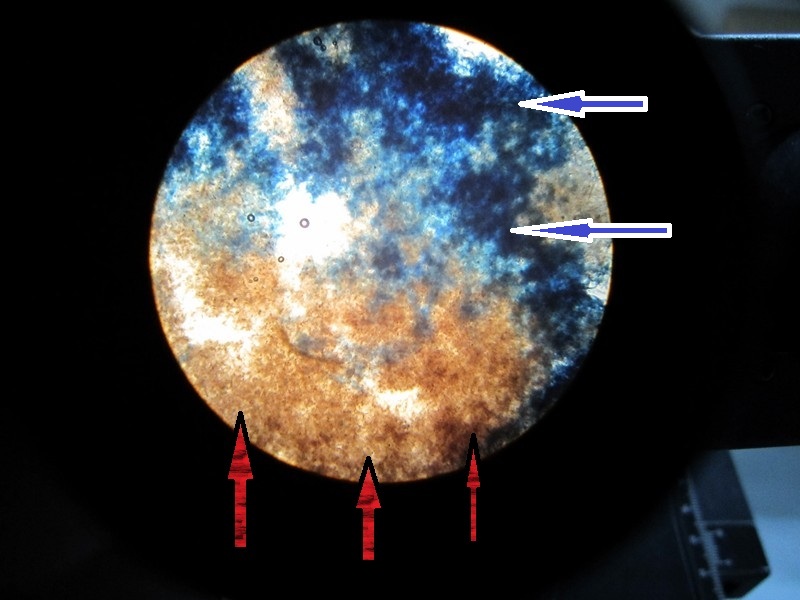
**

Embryogenic cells Non-embryogenic cells

Histological section cells obtained by double staining of cells in morphogenic mass obtained in auxin (left) and cytokinin (right)


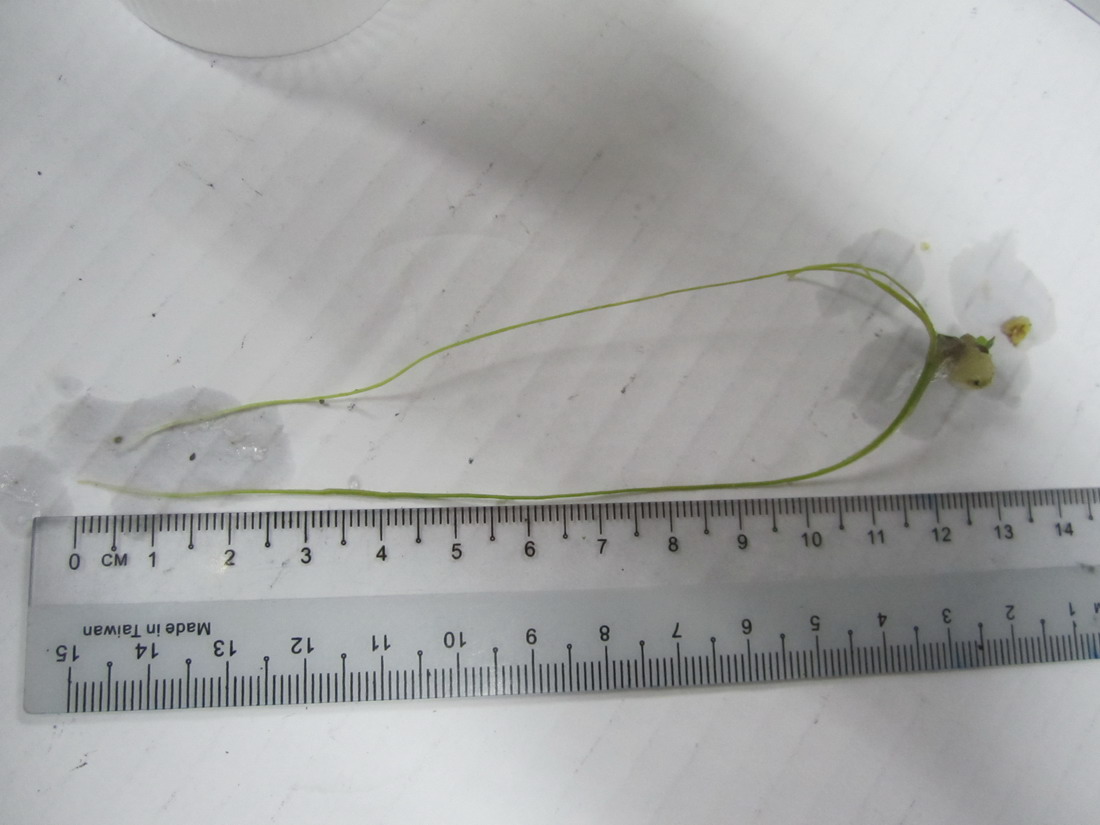


Root formation from lead explant of *G.jasminoides* in MS supplemented with (2 mg L^-1^) NAA after 6 months


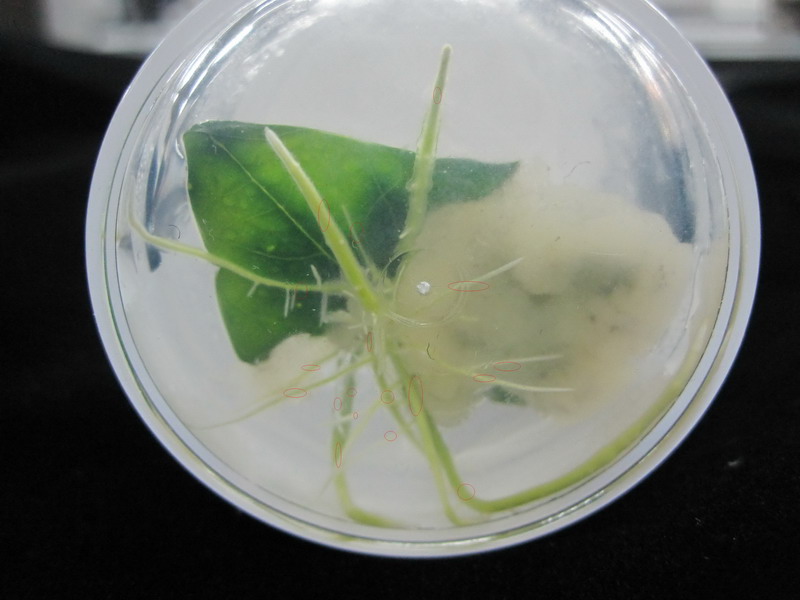


Root elongation from lead explant of *G.jasminoides* in MS media supplemented with (2 mg L^-1^) NAA after 4 months


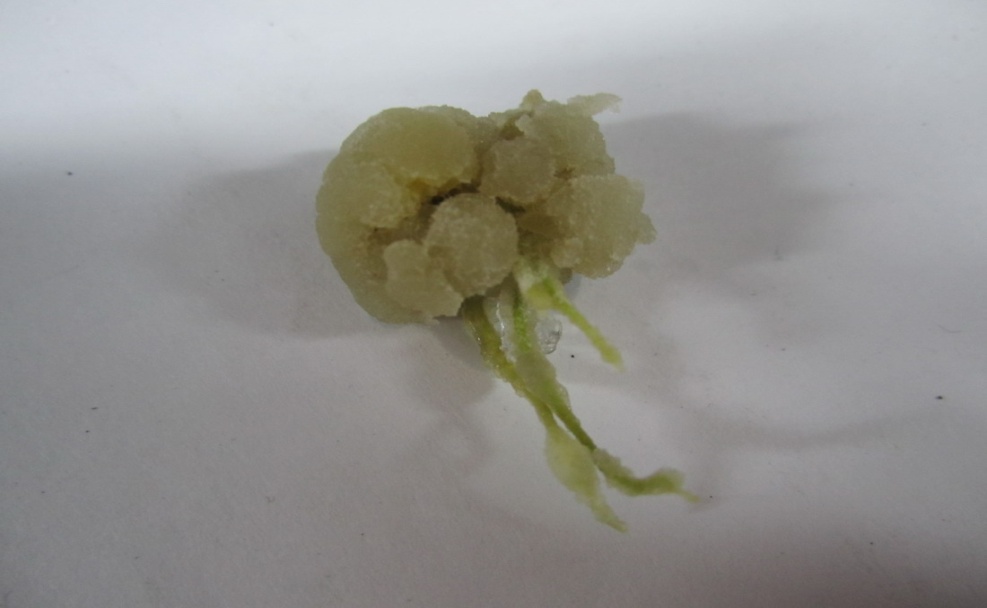
Callus induction and root formation from leaf explant of *G.jasminoides* on MS media supplemented with (1.5 mg L^-1^) NAA after 3 month

**
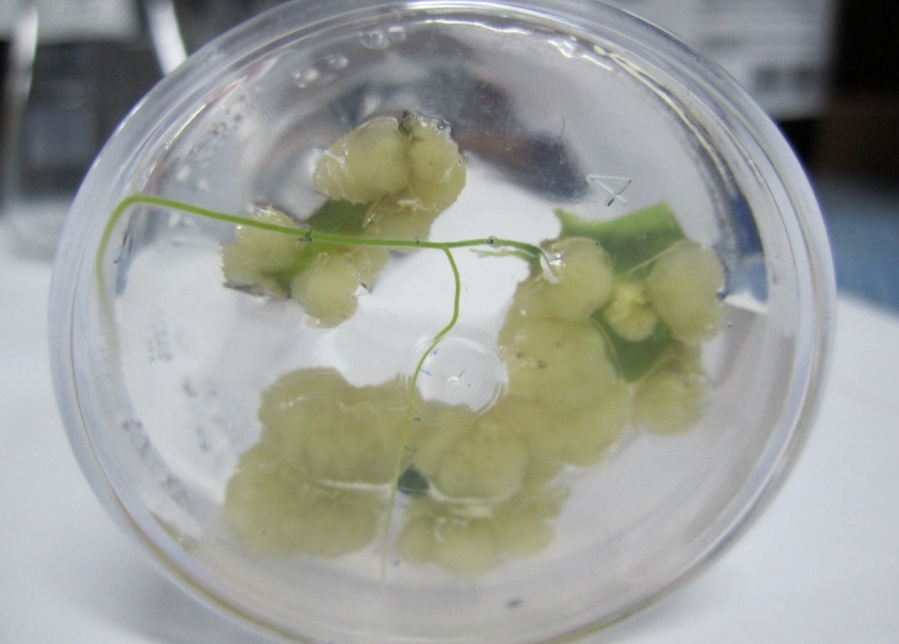
**

Root formation of lead explant from *G.jasminoides* in WPM supplemented with (2 mg L^-1^) IAA after 4 weeks of the second subculture

**
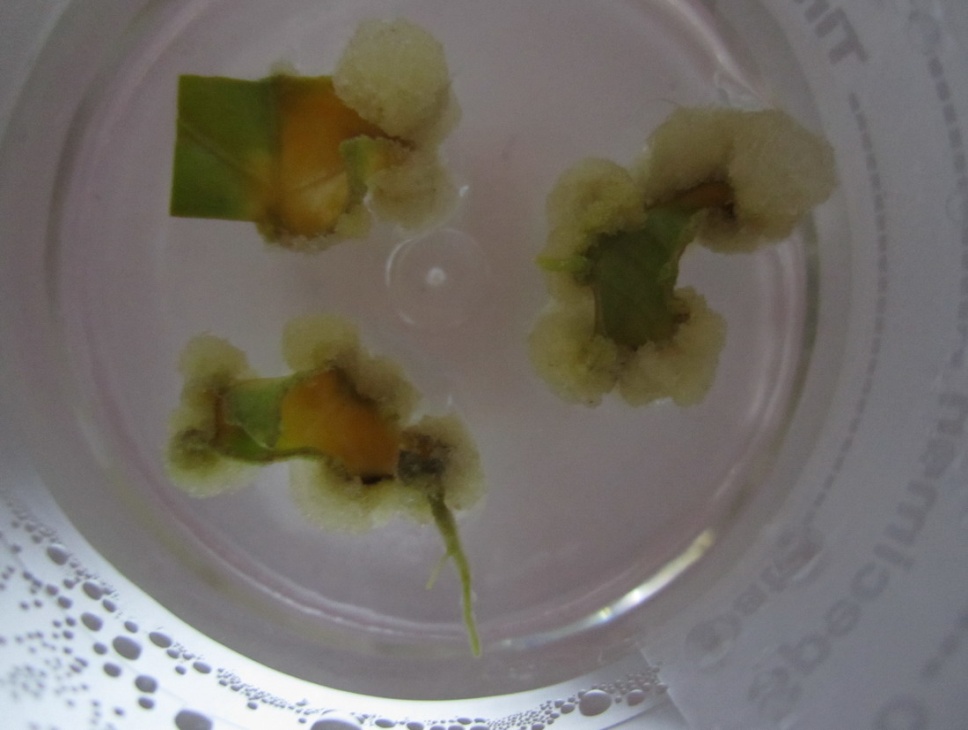
**

Callus induction from leaf explant of *G.jasminoides* in MS medium supplemented with (2.5 mg L^-1^) NAA after 3 months


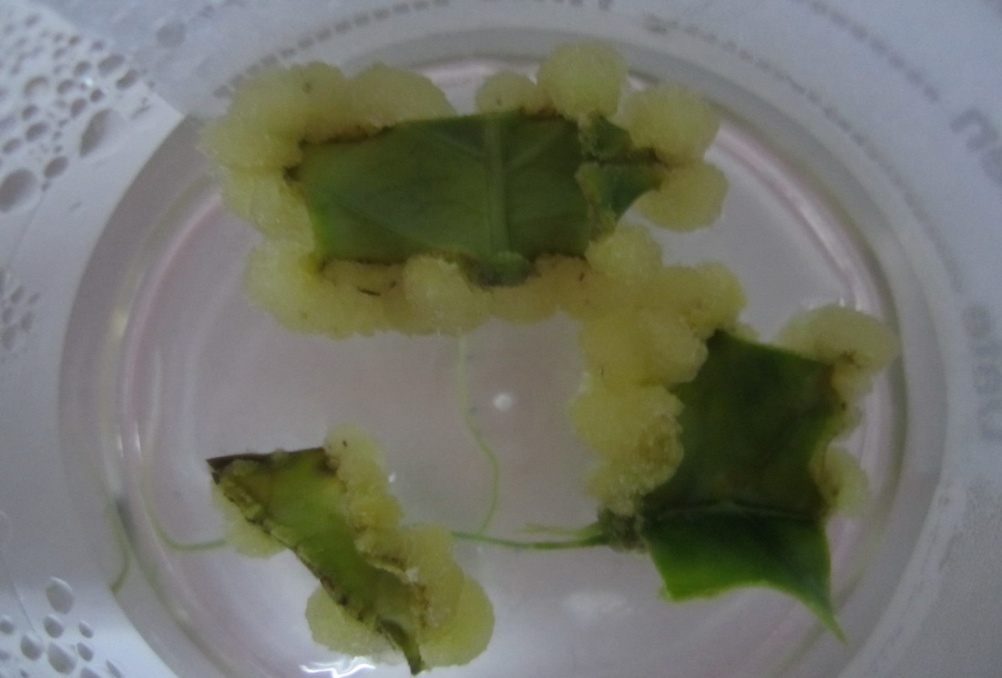


Callus induction from leaf explant of *G.jasminoides* in WPM medium supplemented with (2.5 mg L^-1^) NAA after 3 months


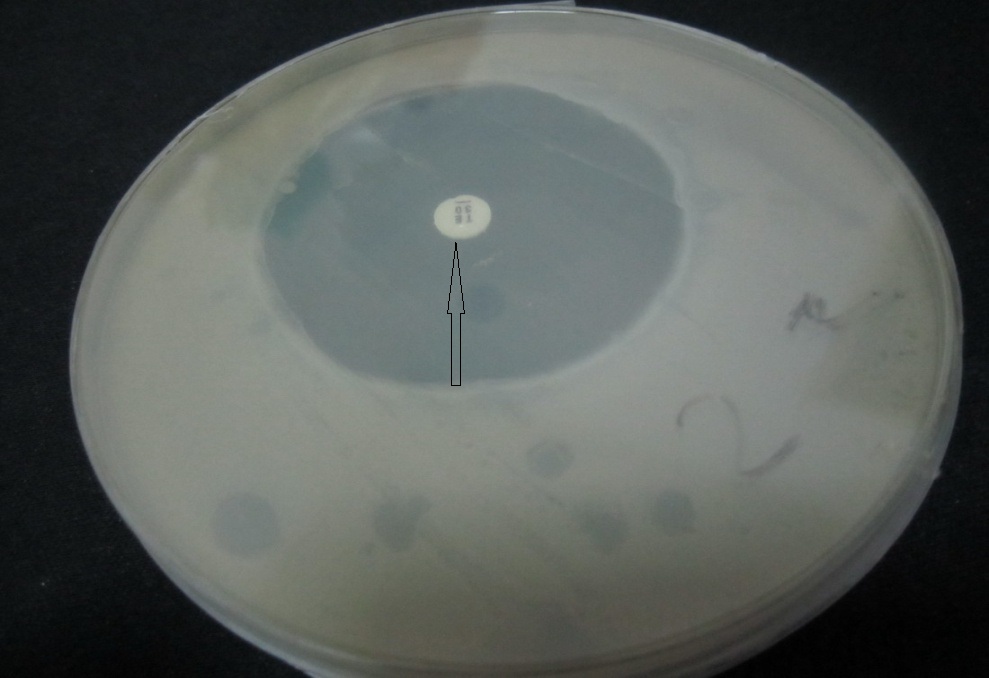


Zone of inhibition of tetracycline as a control


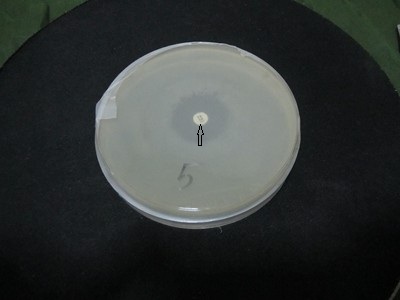


Inhibition zone of *in vitro* extract grown on MS medium supplemented with NAA against *E. coli*


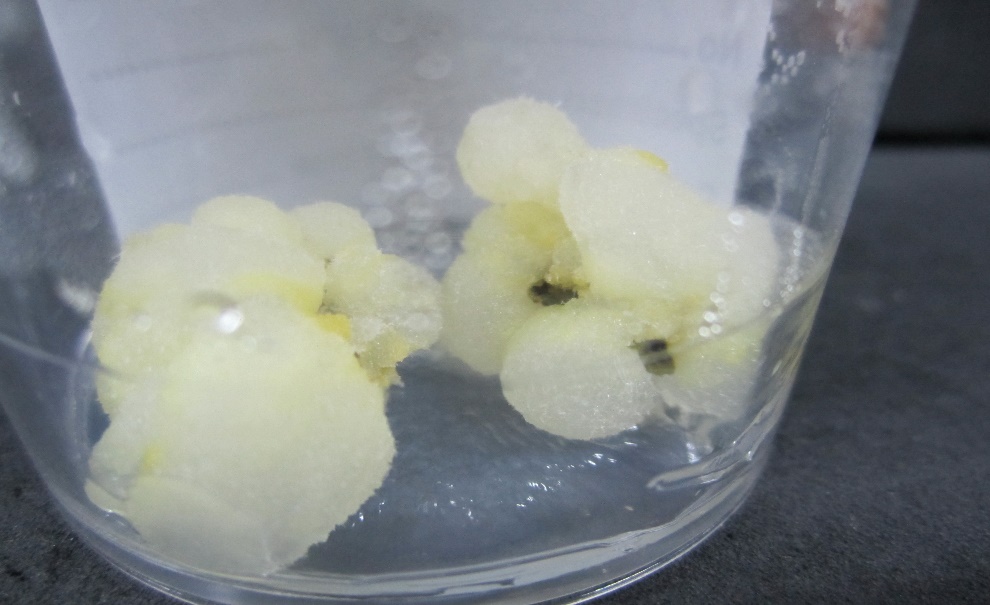

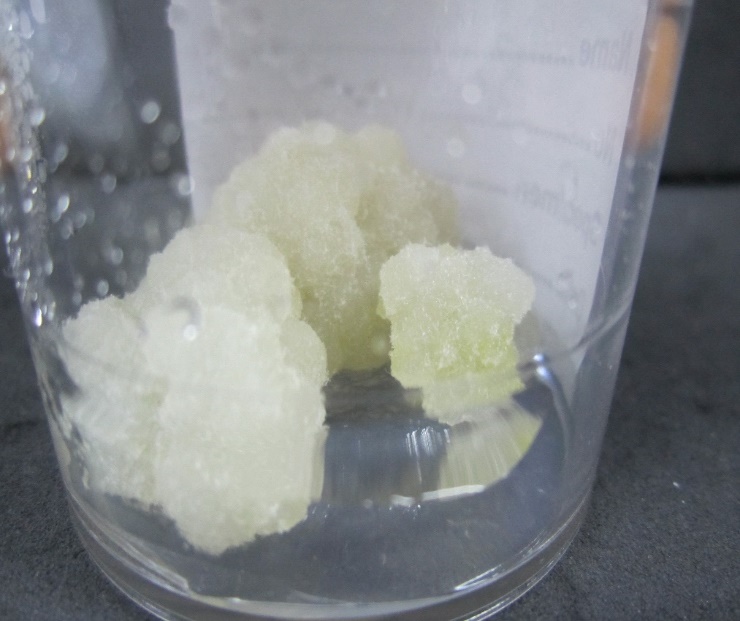


The friable callus from leaf explant of *G.jasminoides* in WPM liquid medium supplemented with (2.5 mg L^-1^) 2,4-D after 12 weeks (left)

The friable callus from leaf explant of *G.jasminoides* in WPM liquid medium supplemented with (2.5 mg L^-1^) NAA after 12 weeks (right)
